# Progress of Obstetrics

**Published:** 1875-05

**Authors:** E. Warren Sawyer

**Affiliations:** Lecturer on Obstetrics, Rush Medical College, Chicago


### Progress in Medical Sciences.

ARTICLE I.

### PROGRESS OF OBSTETRICS.

BY E. WARREN SAWYER, M.D.,

LECTURER ON OBSTETRICS, RUSH MEDICAL COLLEGE, CHICAGO.

1. Gum Cutting. By C. E. BUCKINGHAM, M.D., Professor of Obstetrics, Harvard Medical School. (*Boston Medical and Surgical Journal*, January 21, 1875.)

2. Cephalhæmatoma.

3. Vicarious Menstruation.

4. Mammary Abscess in very young Children.

5. Suspended Animation in New Born Children. (*Boston Medical and Surgical Journal*, February 18, 1875.

6. Medico-Legal Bearing of Puerperal Fever.

1. The writer is surprised to learn, on inquiry, that many of the younger practitioners never use the gum lancet. The "let-alone system of treatment" is sometimes the best, but not in all cases, for the relief which often follows the lancing of the gum "has been more marked than that afforded by any other operation."

Almost all disturbances of the nervous system may be induced by the process of dentition; and many of these disturbances are dissipated by the free lancing of the gum. The relief which follows the loss of a few drops of blood is especially noticed in those cases where the gums are hot and swollen and the mouth is dry.

If it is a molar which is being erupted, a crucial incision is better than a longitudinal one.

Two objections against gum cutting are often raised by "maiden ladies, who are often advisers of mothers younger than themselves": First, that the scar which will result from the incision will be a greater obstacle to the progress of the tooth than the uncut gum. In answer to this, the writer says he has never seen a case in which, if the lancet went well through the gum, down upon the tooth, there was any trouble afterward with that tooth. Even should the gum close, and the indications for cutting recur, the reis no reason why the gum may not be cut again and again.

The second objection is the pretended fear of hæmorrhage. Though instances of grave hæmorrhage from gum cutting are often indirectly heard of, the writer has never yet seen an instance of hæmorrhage, or ever met with a person who had seen hæmorrhage from gum cutting, though the case may be assumed of a bleeder, so called, who might bleed to death from a cut gum, as he would also from a scratch upon the skin.

Finally, the writer believes that infant lives have been saved by timely interference; on the other hand, lives have been sacrificed by withholding this safe and simple operation.

2. At a late meeting of the Obstetrical Society of Boston, Dr. Fifield remarked that he had always been in the habit, as had his father before him, of treating the bloody tumors of the scalp with a lancet, and no harm had ever resulted from the method.

3. At the same meeting, Dr. Jackson stated that he had recently heard of a case in which the blood oozed from the cheek. Dr. Minot spoke of a woman who repeatedly menstruated from the nipples in large quantities.

4. In the number 44, 1874, of *Le Progres Medical*, M. H. Duret has referred to the late controversy upon this subject in the *British Medical Journal*. Dr. Duret cites an instance under his own observation, of an infant of about six weeks old, whose right breast became inflamed and suppurated; strangely enough, the right breast of the mother also suppurated at the same time. This coincidence leads the writer to ask what relation there was between the affection of the mother and child? Is it necessary to go back to a period anterior to the accouchement, when the blood of the mother circulated within the vessels of the fœtus?

Independently of the inflammation and suppuration of the breast, which is excited by the manipulations of the breast by some English nurses, in their attempts "to break the baby's nipple strings," a practice which Dr. Duret properly calls a *contume sauvage*, the breasts of very young infants are sometimes the seat of an inflammation, and suppuration, which may extend to the axilla, or mount even to the side of the neck.

5. This was the subject of a recent lecture at the Harvard Medical School, by Prof. Buckingham. In the blue and flabby child, even if pulsations have ceased in the cord, you may hear the fœtal pulsations over the heart. Do not stop to tie the cord; let it bleed a little. Send for two pails of water, the one cold and the other a little too warm to bathe in; a child who has never attempted to breathe will often cry lustily after being plunged alternately into the cold and warm bath. But before the water has reached you, try to inflate the lungs, in this manner: first wipe the mucus from the child's mouth; take the child up in a towel so it will not slip from your grasp; hold it with the scapulæ in the left palm; extend the head fully, so as to close the œsophagus, and seize the occiput between the thumb and index finger of the left hand; apply your mouth closely to that of the child, and inflate the lungs with your breath. There is no danger of your blowing too hard ; "indeed, unless you place a moderately dry cloth between the child's mouth and your own, you will find it difficult to inflate at all." The lungs should be inflated ten or fifteen times per minute, meanwhile alternating your efforts with the plunges into the baths. Do not relinquish your exertions till all evidences of life have disappeared.

When the child is resuscitated, place it unwashed in a blanket, without food, where it will be better, for the next twenty-four hours, than if annoyed with any further attentions.

6. The February number of the *Obstetrical Journal of Great Britain* contains an account of a midwife, a Mrs. Ingram, who had been unfortunate in meeting with a number of cases of puerperal fever, some of which had died. The coroner, after examining into the cause of death of one of her patients, notified her that she should not again practice midwifery until two medical men had certified that she might safely do so. In defiance of this warning, she afterward attended upon two women in labor, both of whom died from puerperal fever. The coroner's jury rendered a verdict of manslaughter. She was committed in default of bail, which was afterwards furnished in sureties of £200, and she was released till the next assizes at Warwick. The Journal continues: "There are several points of interest in this case. First, the question as to the contagiousness of puerperal fever, the reality of which some individuals might perhaps be found to dispute ; second, the legal right of a coroner to step in and warn an obstetrician not to attend women in labor ; and, thirdly, does not it again demonstrate the necessity of instructing midwives ? Mrs. Ingram's attainments are sufficiently manifested in her answer to the coroner, who asked her if she knew what child-bed fever was: 'I never met with it, and did not even know till Dr. Brown told me. I never had a woman done badly in my life before—never.' "

